# Scaling of oscillatory kinematics and Froude efficiency in baleen whales

**DOI:** 10.1242/jeb.237586

**Published:** 2021-07-09

**Authors:** William T. Gough, Hayden J. Smith, Matthew S. Savoca, Max F. Czapanskiy, Frank E. Fish, Jean Potvin, K. C. Bierlich, David E. Cade, Jacopo Di Clemente, John Kennedy, Paolo Segre, Andrew Stanworth, Caroline Weir, Jeremy A. Goldbogen

**Affiliations:** 1 Hopkins Marine Station, Stanford University, Pacific Grove, CA 93950, USA; 2 Department of Physics, Southwestern University, Georgetown, TX 78626, USA; 3 Department of Biology, West Chester University, West Chester, PA 19383, USA; 4 Department of Physics, Saint Louis University, Saint Louis, MO 63103, USA; 5 Nicholas School of the Environment, Duke University, Durham, NC 27708, USA; 6 Long Marine Laboratory, University of California Santa Cruz, Santa Cruz, CA 95064, USA; 7 Department of Biology, University of Copenhagen, Copenhagen, Denmark; 8 Falklands Conservation, Stanley FIQQ 1ZZ, Falkland Islands

**Keywords:** Cetacean, Swimming, Hydrodynamics, Thrust, Efficiency

## Abstract

High efficiency lunate-tail swimming with high-aspect-ratio lifting surfaces has evolved in many vertebrate lineages, from fish to cetaceans. Baleen whales (Mysticeti) are the largest swimming animals that exhibit this locomotor strategy, and present an ideal study system to examine how morphology and the kinematics of swimming scale to the largest body sizes. We used data from whale-borne inertial sensors coupled with morphometric measurements from aerial drones to calculate the hydrodynamic performance of oscillatory swimming in six baleen whale species ranging in body length from 5 to 25 m (fin whale, *Balaenoptera physalus*; Bryde's whale, *Balaenoptera edeni*; sei whale, *Balaenoptera borealis*; Antarctic minke whale, *Balaenoptera bonaerensis*; humpback whale, *Megaptera novaeangliae*; and blue whale, *Balaenoptera musculus*). We found that mass-specific thrust increased with both swimming speed and body size. Froude efficiency, defined as the ratio of useful power output to the rate of energy input ( Sloop, 1978), generally increased with swimming speed but decreased on average with increasing body size. This finding is contrary to previous results in smaller animals, where Froude efficiency increased with body size. Although our empirically parameterized estimates for swimming baleen whale drag were higher than those of a simple gliding model, oscillatory locomotion at this scale exhibits generally high Froude efficiency as in other adept swimmers. Our results quantify the fine-scale kinematics and estimate the hydrodynamics of routine and energetically expensive swimming modes at the largest scale.

## Abstract (precis)

**Summary:** Tags attached to baleen whales demonstrate how thrust power output, drag coefficient and Froude efficiency scale with swimming speed and body length.

## INTRODUCTION

The repeated invasion of aquatic and marine environments by tetrapods over the last 250 million years has resulted in a host of convergent morphological adaptations that facilitate life in water (Kelley and Pyenson, 2015). Among these adaptations are the evolution of a fusiform body shape, flattened control surfaces and sickle-shaped caudal fin to achieve high performance locomotion (Fish et al., 2008). These morphological adaptations are functionally analogous among swimming animals such as thunniform fish, lamnid sharks, cetaceans and the extinct ichthyosaurs (Motani, 2002; Donley et al., 2004; Gleiss et al., 2011). The majority of these swimmers use an oscillatory swimming style that involves side-to-side or up-and-down movement of a hydrofoil-like tail to generate lift-based thrust and overcome drag (Fish, 1998). Cetaceans are unique among oscillatory swimmers because of their extreme body mass, exemplified in modern baleen whales (Mysticeti), which evolved massive body sizes within the last five million years (Slater et al., 2017).

Although the swimming performance of large whales has long been of interest to researchers (Krogh, 1934; Kermack, 1948; Bose and Lien, 1989), direct measures of their swimming kinematics and morphology have been difficult to obtain. Studies of cetacean swimming kinematics have typically focused on smaller and highly maneuverable odontocete species in captivity (Fish, 1993, 1998; Curren et al., 1994; Fish et al., 2014). Attempts to study mysticetes and derive energetic assumptions (Sumich, 1983; Parry, 1949; Blix and Folkow, 1995) were constrained to breathing events at the water's surface, and morphological measurements were only attainable from deceased animals that had stranded on beaches or had been captured by whaling operations (Lockyer, 1976; Kahane-Rapport and Goldbogen, 2018). The recent development of high-resolution biologging methods now allows researchers to quantify the kinematics of free-swimming cetaceans in their natural habitats (Johnson and Tyack, 2003; Goldbogen et al., 2017a; Gough et al., 2019). In addition, unoccupied aircraft systems (UAS, or drone) technology has enhanced our ability to obtain precise morphological data, thereby enabling comparative and scaling analyses of form and function (Gough et al., 2019; Christiansen et al., 2019; Kahane-Rapport et al., 2020).

**Fig. 1.**
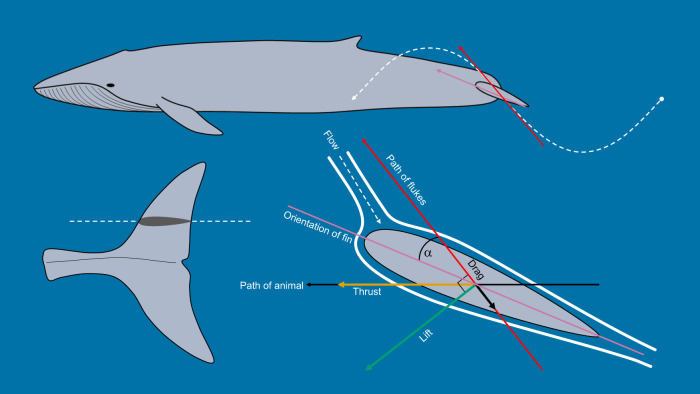
**Adaptation from** **Shadwick (2005)** **showing the forces acting on the tail of a thunniform swimmer such as a blue whale during active oscillatory fluking of the tail.** The heaving motion of the tail creates a pressure imbalance between the top and bottom faces of the fluke that results in the generation of a lift force perpendicular to the path of the flukes and a thrust force in the forward direction of travel of the animal.

Understanding the size-dependent kinematics of swimming cetaceans is critical to analyze their swimming performance and energetics. The dorso-ventral oscillation of the flukes produces lift that is resolved into a forward thrust vector (Fig. 1) (Lighthill, 1971; Chopra and Kambe, 1977; Vogel, 1994; Fish, 1998). This lift-based thrust power is equal to the drag power of the animal when swimming at a constant velocity (Lighthill, 1971; Fish, 1998). This mechanism is considered to be highly efficient (>75%; Triantafyllou et al., 1991; Rohr and Fish, 2004). Previous attempts to estimate the thrust power of actively swimming large whales have been made based on a number of assumptions without reliable kinematic data (Parry, 1949; Chopra and Kambe, 1977; Yates, 1983; Bose and Lien, 1989). Thrust power generation is modulated through the adjustment of basic kinematic parameters of the oscillatory tailbeat cycle, and new biologging tags make these empirical measurements possible for large, free-swimming animals.

Kinematic studies performed on cetaceans have focused on the three fundamental parameters of an oscillatory tailbeat cycle: amplitude of heave, swimming speed, and oscillatory frequency. Among these, speed has been studied most extensively. Using various methods, researchers have found that many different species of cetaceans are able to swim over an extended range of speeds. High speeds in excess of 8 m s^−1^ have been achieved by rorqual mysticetes (Fish and Rohr, 1999; Hirt et al., 2017; Segre et al., 2020). A recent study by Gough et al. (2019) has shown that mysticetes tend to swim at ∼2 m s^−1^ when not feeding. In order to swim at different speeds within this wide range, mysticetes must adjust either their oscillatory frequency or the amplitude of heave (Lighthill, 1971; Chopra and Kambe, 1977). For small odontocetes, Fish (1998) found that oscillatory frequency increased with increasing swimming speed but decreased roughly with body length, while amplitude of heave remained constant at ∼0.2 of an animal's body length. These findings were recently confirmed for mysticetes by Gough et al. (2019).

Measuring the fundamental kinematic parameters of the oscillatory tailbeat cycle has allowed researchers to estimate Froude efficiency, or the percentage of thrust that is successfully transferred into forward motion (Vogel, 1994; Fish, 1998). The dimensionless Strouhal number has typically been used as a rough way to describe how the amplitude of heave, swimming speed, and oscillatory frequency are modulated and interact to provide a maximally efficient pattern of vorticity around the tail during swimming (Triantafyllou et al., 1991; Fish, 1998; Taylor et al., 2003; Rohr and Fish, 2004; Gough et al., 2019). The generally accepted rule is that highly efficient oscillatory swimming falls within a Strouhal range of 0.25 to 0.35 (Triantafyllou et al., 1991). Both Rohr and Fish (2004) and Gough et al. (2019) found that cetaceans fall within this range, but a more detailed analysis of the kinematics and hydrodynamic parameters, such as the thrust power output and drag, has only been performed previously by Fish (1998) for much smaller odontocetes.

Here, our goal was to move beyond the Strouhal number and use a combination of whale-borne tags and UAS morphological measurements to calculate the kinematics, thrust power output and Froude efficiencies for free-swimming mysticete whales using methods similar to Fish (1998). Apart from Gough et al. (2019), we have a very limited understanding of how kinematics affect swimming performance at the upper extremes of body size. Previous studies have estimated the Froude efficiency of swimming for odontocetes and other oscillatory swimming animals to be approximately ∼75–90% (Fish, 1998), but the only estimate for a mysticete prior to our study came from a single fin whale (*Balaenoptera physalus*) of unknown body size swimming at ∼8 m s^−1^ (Bose and Lien, 1989). Our current data set goes beyond any previous analyses and includes six species and a ∼5× range in body length. All of the species included in our study are lunge feeders, which open their mouth wide prior to engulfing a large volume of water into a highly expansible throat pouch (Goldbogen et al., 2017b). This behavior requires the efficient achievement of high swimming speeds in order to maintain a favorable energetic balance (Potvin et al., 2009, 2020, 2021). We hypothesize that the kinematic and hydrodynamic parameters of swimming scale similarly between small and large cetaceans and will lead to high (>75%) Froude efficiencies for even the largest animals. Our study will lead to a more complete scaling-based understanding of oscillatory swimming in mysticetes and the kinematic, hydrodynamic and morphological factors that impact swimming performance in the world's largest animals.

## MATERIALS AND METHODS

### Study species and locations

The whales included in this study are the Antarctic minke whale (*Balaenoptera bonaerensis* Burmeister 1867), humpback whale (*Megaptera novaeangliae* Borowski 1781), fin whale [*Balaenoptera physalus* (Linnaeus 1758)], Bryde's whale (*Balaenoptera edeni* Anderson 1879), sei whale (*Balaenoptera borealis* Lesson 1828) and blue whale [*Balaenoptera musculus* (Linnaeus 1758)]. The six species are members of the family Balaenopteridae, commonly referred to as rorquals, and tend to have similar life histories and behaviors. These species range in size from ∼5 m in length for the Antarctic minke whale up to ∼25 m for an adult blue whale (Goldbogen et al., 2019). Distinct morphological differences are also present between these species (Kahane-Rapport and Goldbogen, 2018), with the most prominent being the enlarged flukes and flippers of the humpback whale relative to body size (Fish and Battle, 1995; Woodward et al., 2006).

Data on foraging and swimming were collected on humpback whales off of the coast of Monterey, CA, USA, and the Western Antarctic Peninsula, blue whales off California (Monterey Bay and Southern California Bight), Antarctic minke whales off the western Antarctic Peninsula, fin whales in Monterey Bay and the fjords of southeastern Greenland, Bryde's whales off the southern coast of South Africa, and sei whales near the Falkland Islands. All work was performed under suitable permits and in accordance with university IACUC procedures. All procedures in the USA and Antarctica (permit 14809) were conducted under approval of the National Marine Fisheries Service (permits 781-1824, 16163, 14809, 16111, 19116, 15271, 20430); elsewhere, procedures were conducted under approval of Canada DFO SARA/MML 2010-01/SARA-106B, National Marine Sanctuaries (MULTI-2017-007), research permit R23.2018 issued by the Falkland Islands Government, ACA 2015-014, and institutional IACUC committees.

### CATS tags

The Customized Animal Tracking Solutions (CATS) tags integrate video with 400 Hz accelerometers and gyroscopes; 50 Hz magnetometers, pressure and temperature sensors; a 10 Hz internal temperature sensor; and 10 Hz light and GPS sensors. Tag accelerometers for all whales were sampled at 40 or 400 Hz, magnetometers and gyroscopes at 40 or 50 Hz, and pressure, light, temperature and GPS at 10 Hz. All data were decimated to 10 Hz, tag orientation on the animal was corrected for, and animal orientation was calculated using custom-written scripts in MATLAB 2014a (following Johnson and Tyack, 2003; Cade et al., 2016). Animal speed for all deployments was determined using the amplitude of tag vibrations, a method that has been shown to be robust and accurate above ∼1 m s^−1^ in a variety of behavioral contexts (Cade et al., 2018). The tags were deployed from rigid-hull inflatable boats using a 6 m carbon-fiber pole. These attached to the animal via four suction cups, detached after suction failed, floated to the surface and were recovered via VHF telemetry. Deployment lengths in this study ranged from 8 min to 26 h. For more information on the type of tag used in this study, see Goldbogen et al. (2017a).

### UAS operations and morphometric measurements

Images of each species were collected using UAS between 2017 and 2019. Specifically, two types of stock-build quadcopters, the Phantom 3 and Phantom 4 Pro, as well as two types of custom hexacopters were used, the FreeFly Alta 6 and a Mikrokopter-based LemHex-44. Both quadcopters used stock-built barometers and cameras while the hexacopters contained a two-axis gimbal fitted with a Lightware SF11/C laser altimeter and a Sony Alpha A5100 camera with an APS-C sensor (23.5×15.6 mm), 6000×4000 pixel resolution, and either a Sony SEL 50 mm or SEL 35 mm focal length low distortion lens.

**Fig. 2.**
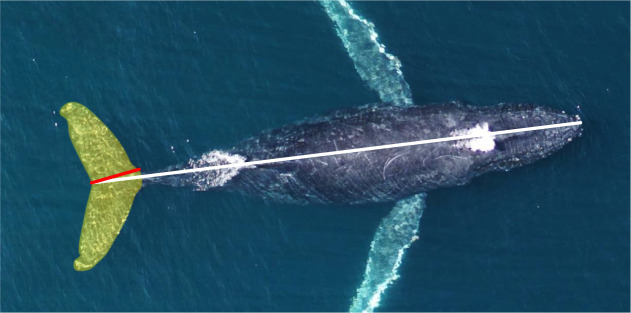
**Representative UAS drone image of a humpback whale showing the morphometric measurements taken from each animal.** The white line corresponds to the total length (in meters) from the tip of the lower jaw to the caudal midpoint of the tail. The chord length of the fluke (in meters) is denoted by the red line running from the cranial insertion of the fluke onto the peduncle to the caudal midpoint of the tail. The light orange shaded region corresponds to the tail area (in m^2^) comprising the entirety of the flukes and the peduncle region caudal to the cranial fluke insertions.

ImageJ 1.5i (Schindelin et al., 2012) was used to measure the total length, maximum body diameter, fluke chord length and fluke area (Fig. 2). Measurement errors for each aircraft were estimated by measuring a known-sized object floating at the surface from various altitudes, and each aircraft had an average altitude error of <5%. Measurements in pixels were multiplied by the ground sampling distance (GSD) to convert to meters following Fearnbach et al. (2012):(1)
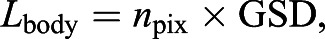
(2)
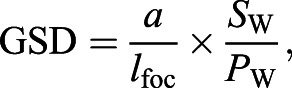

where *L*_body_ is the body length (m), *n*_pix_ is the number of pixels, *a* is the altitude (m), *l*_foc_ is the focal length (mm), *S*_w_ is the sensor width (mm) and *P*_w_ is the image resolution width (pixels). (All equation symbols used in this article are also listed in Table S1.) The width of the sensor and image resolution was used because images of the whales were captured full frame widthwise (Gough et al., 2019). In ImageJ (National Institutes of Health), the combined planar surface area of the flukes (*F*_a_; m^2^) was calculated by carefully drawing a polygonal outline of the flukes. Chord length of the flukes (*C*; m) was measured as the linear distance from the notch between the flukes to the anterior insertion of the flukes on the tail. Body mass (*M*_body_; kg) was estimated from total body length using regressions derived for each of our six study species using a broad range of data compiled from both whaling operations and studies of stranded animals (Kahane-Rapport and Goldbogen, 2018). The wetted surface area of the body (*S*_a_; m^2^) was estimated from total body length using equations derived from various sources and summarized in Table S2.

### Routine and lunge-associated tailbeat detection

We used a customized MATLAB script to detect tailbeat cycles based upon methods defined by Gough et al. (2019). In particular, a series of thresholds was used to define periods in the filtered (low-pass; 0.44 Hz) gyroscope signal (along the transverse axis) corresponding to individual tailbeats. These thresholds checked for symmetry between the upstroke and downstroke by defining the magnitude, duration and overall shape of each portion of the tailbeat cycle. The resulting set of tailbeat cycles was spot-checked and compared against tag video to ensure that the parameters were set correctly. Individual whales must have had a dataset of >200 tailbeats in order to be included for further analysis.

Foraging lunges were detected manually using a series of defined kinematic parameters that have been validated using tag video (Cade et al., 2016). These events typically involve an increase in speed during prey approach, followed by a rapid deceleration as an animal opens its mouth to engulf prey (Potvin et al., 2009; Goldbogen et al., 2011; Cade et al., 2016; Potvin et al., 2021). We standardized the period from 10 to 0 s prior to the lunge deceleration (which typically coincides with the period of mouth opening) as the lunge-associated period. This length of time corresponds to the approximate length of the acceleration period for a minke whale and the duration of two cruising tailbeats for a blue whale. By choosing this period immediately prior to the lunge for each species in our dataset, we can capture full tailbeats that display high swimming speeds, but a fully closed mouth and hydrodynamic profiles similar to those of routine swimming. We observed that whales do not commonly fluke with their mouth open or during subsequent filtration, but we explicitly excluded any tailbeats during these periods to avoid high drag from the distended throat pouch. Any tailbeat that occurred within the lunge-associated time period was classified as lunge-associated. All other tailbeats were classified as routine swimming. The lunge-associated tailbeats included a greater change in swimming velocity, but our tailbeat detection thresholds ensured general consistency in the overall kinematic profile of the tailbeats and resulted in two sets of tailbeats at different levels of swimming effort.

### Thrust power, efficiency and drag coefficient modeling

For each routine and lunge-associated tailbeat, we measured the mean swimming velocity (*U*_avg_; m s^−1^) by averaging across the entire time course of the cycle. Because the measurement of speed by the tag required turbulent flow, speed measurements were limited to >1 m s^−1^ (Cade et al., 2018). We also measured oscillatory frequency (*f*; Hz) as the inverse of the duration of the tailbeat cycle (*T*_beat_; s). For routine tailbeats, we calculated (mechanical) thrust power (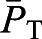; W), coefficient of drag (*C*_D_) and Froude efficiency (η) based on a model of lunate tail propulsion using unsteady wing lifting surface theory (Chopra and Kambe, 1977; Yates, 1983; Fish, 1998). This model begins with the estimation of two input parameters, namely, the reduced frequency (σ) defined as:(3)
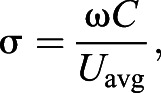


where ω is the angular frequency of fluking (with ω*=*2π*f*); and the feathering parameter (ω) defined as:(4)
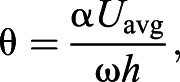


which is expressed as the ratio of the maximum angle (α; deg) between the fluke and the direction of motion and the maximum angle (ω*h*/*U*) achieved by the trajectory of the pitching axis of the flukes (Yates, 1983) when reaching the heave amplitude (*h*; m). We were unable to measure precise values for α or *h* from the tag data and instead relied on validated estimates of 30 deg for α and one-fifth of body length for *h* (Bainbridge, 1958; Fish, 1998).

The model devised by Chopra and Kambe (1977) yielded a series of parametric curves expressing the coefficient of thrust (*C*_T_) and Froude efficiency in terms of σ and ω (Yates, 1983). We digitized these curves and estimated both values for each tailbeat cycle, and then estimated the mean thrust force (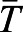; *N*) (over a tailbeat cycle) and corresponding mean thrust power (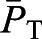) as follows (Fish, 1993):(5)
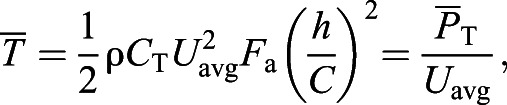


where ρ is the density of seawater (Table S1). Previous versions of this model assumed steady-state swimming during which the energy gained through propulsion (thrust) matches what is lost through drag, an equality from which the drag coefficient could be obtained (Fish, 1993, 1998). Given the high speed variability inherent in natural tail-heaving swimming, the relationship between mean thrust and mean drag had to be re-written to account for the body's forward acceleration or deceleration during a tailbeat. We started with the equation of motion of the body averaged over the duration of a beat, namely:(6)
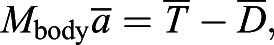


where the mean acceleration is given by 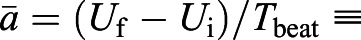 
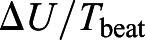
, with *U*_f_ as the final speed at the end of the tailbeat, *U*_i_ the initial speed at its beginning, and *T*_beat_ as its duration. Given the high degree of body streamlining, the mean drag force (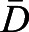; *N*) is expressed as follows (Goldbogen et al., 2019; Potvin et al., 2020; Segre et al., 2020; Potvin et al., 2021):(7)
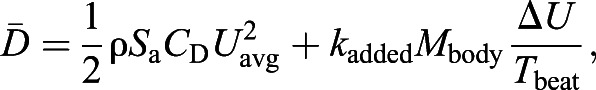


a result involving the corresponding ‘mean drag coefficient’ across the duration of the tailbeat (*C*_D_). The parameter *k*_added_ is an acceleration reaction coefficient set at 0.03 for blue whales and minke whales and 0.05 for humpback whales (Potvin et al., 2020, 2021). Merging Eqns 5–7 and solving for the drag coefficient results in:(8a)
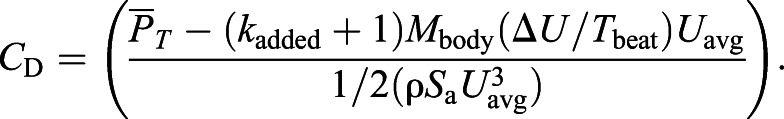


In this formulation, the tag-measured beat duration (*T*_beat_) and change in speed (Δ*U*) quantifies, via the second term in the equation, the effects on the drag coefficient of unsteadiness in a whale's forward speed. Setting it to zero recovers the familiar steady-state case.

For each whale, we found the mean drag coefficient across all routine tailbeats (*C*_D,routine_) and used that value to estimate the mean thrust power (
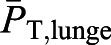
; W) for each lunge-associated tailbeat. This calculation involved reordering Eqn 8a to solve for the mean thrust power:(8b)
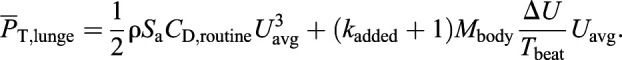


As a final note, it should be mentioned that estimating the thrust via Eqn 5 and the graphs found in Yates (1983) represents the closest approximation possible at the present time.

### Comparison to a simple rigid-body model

Cetacean swimming involves body and tail heaving motions that are altogether absent with the motions of rigid bodies (e.g. submarines) and significantly increase drag (Fish, 1993, 1998; Fish and Rohr, 1999). We compared our drag coefficient data with that of airship models tested in wind tunnels (and at constant wind speed), as correlated by the following equation (Hoerner, 1965; Webb, 1975; Blevins, 1984; Kooyman, 1989):(9)
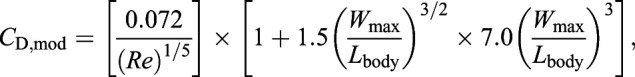
where *C*_D,mod_ is the modeled drag coefficient and *W*_max_ is the maximum body diameter (m). This equation is expressed in terms of the Reynold's number (*Re*):(10)
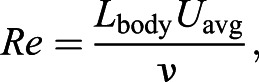


in which ν is the kinematic viscosity. In this case, the drag force (*F*_drag,parasite_) sustained by the airship (or non-tail-heaving whale) is given by:(11)
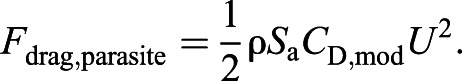


Table S1 contains a list of all symbols used throughout this paper.

### Statistical analyses

For our analyses of mean swimming speed and oscillatory frequency against body length, continuous variables (body length, oscillatory frequency and mean swimming speed) were log_10_ transformed before inclusion as predictors or response variables to normalize our data and conform to the model of scaling as a power function. For these analyses, we created linear mixed-effects models with body length as the predictor, oscillatory frequency and mean swimming speed as response variables, and species as a random effect. For subsequent analyses, we created linear mixed-effects models with body length, mean swimming speed and Reynold's number as predictors, thrust power, drag coefficient and Froude efficiency as response variables, and species as a random effect. These models were created using using R v. 3.6 and RStudio [version 1.2.1335, https://www.r-project.org/; packages ggpubr (https://CRAN.R-project.org/package=ggpubr) and tidyverse (Wickham et al., 2019)]. We fitted linear regressions to assess relationships using package lme4 in R. For our analysis of swimming speed versus Froude efficiency, we used a generalized additive model (GAM) in R (*y*∼*s*[*x*, bs=“cs”]).

## RESULTS

### Kinematic and morphometric summary

We investigated interspecific relationships between 65 animals and found that mean (±s.e.m.) values for oscillatory frequency (Hz) and swimming speed (m s^−1^) both increased when transitioning from routine to lunge-associated swimming. The mean increase in (time-averaged) swimming speed between the two modes was 0.762±0.154 m s^−1^ and the mean increase in oscillatory frequency was 0.102±0.017 Hz (Table 1).

**Table 1.**
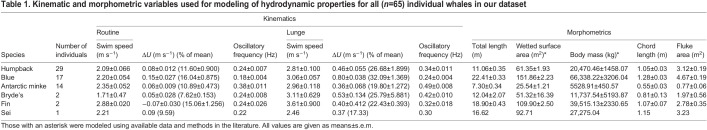
Kinematic and morphometric variables used for modeling of hydrodynamic properties for all (*n*=65) individual whales in our dataset

**Fig. 3.**
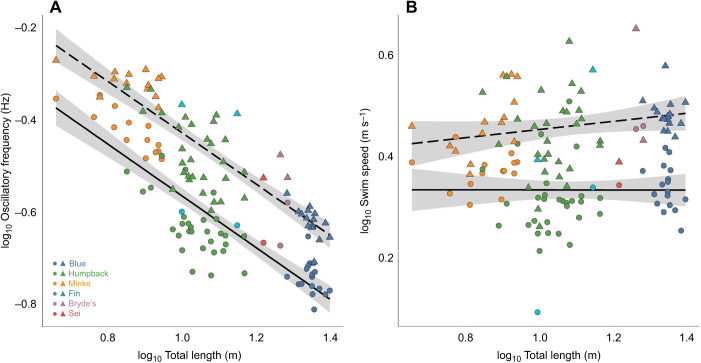
**Comparison of total body length with oscillatory frequency and swim speed for two swimming modes.** Linear regressions showing the log_10_ of total body length (m) versus the (A) oscillatory frequency (Hz) and (B) swim speed (m s^−1^) for both routine swimming (solid line) and lunge-associated swimming (dashed line). Each point corresponds to the mean value for a single individual whale and a single swimming mode (circle: routine; triangle: lunge-associated).

We found that the mean oscillatory frequency for the three species with the most data (humpback, blue, Antarctic minke) decreased with increasing body length, with the Antarctic minke whale having the highest values (routine: 0.38±0.011 Hz; lunge-associated: 0.49±0.008 Hz), followed by the humpback whale (routine: 0.24±0.007 Hz; lunge-associated: 0.34±0.011 Hz) and the blue whale (routine: 0.18±0.004 Hz; lunge-associated: 0.24±0.004 Hz). Bryde's and fin whales had similar routine oscillatory frequencies as the humpback whale, while having longer average body lengths (Bryde's: 12.04±2.07 m; fin: 18.90±0.43 m) than the humpback whales in our study (11.06±0.35 m). Both of the oscillatory frequency values for the lone tagged sei whale (routine: 0.22 Hz; lunge-associated: 0.30 Hz) fell approximately halfway between the values for the humpback and blue whales, which aligns with the sei whale's body length (16.62 m) being approximately halfway between the mean humpback and blue whale (22.41±0.33 m) body lengths. We found significant negative relationships between oscillatory frequency and body size during both routine and lunge-associated swimming (routine: *ŷ*=−0.565*x*+0.003, *R*^2^=0.75, *P*<0.001; lunge-associated: *ŷ*=−0.560*x*+0.312, *R*^2^=0.77, *P*<0.001; Fig. 3A).

The mean values for both routine and lunge-associated swimming speeds were similar for the humpback (routine: 2.09±0.066 m s^−1^; lunge-associated: 2.81±0.100 m s^−1^), blue (routine: 2.20±0.054 m s^−1^; lunge-associated: 3.06±0.057 m s^−1^) and Antarctic minke whales (routine: 2.35±0.052 m s^−1^; lunge-associated: 2.96±0.118 m s^−1^). Despite low sample sizes, the average routine and lunge-associated swimming speeds for the Bryde's whale (routine: 1.71±0.47 m s^−1^; lunge-associated: 3.11±0.629 m s^−1^) and the routine swimming speed for the sei whale (2.21 m s^−1^) aligned with those of the humpback, blue and Antarctic minke whales, while the lunge-associated swimming speed for the sei whale (2.46 m s^−1^) was lower than other values and both swimming speeds were higher for the fin whale (routine: 2.88±0.020 m s^−1^; lunge-associated: 3.61±0.900 m s^−1^). The average routine swimming speed across all species was found to be 2.18±0.001 m s^−1^. The median routine swimming speed across all species was found to be 2.06 m s^−1^. Our statistical analysis found no effect of body size on swim speed for either routine or lunge-associated swimming (routine: *ŷ*=−0.001*x*+0.774, *R*^2^=6.27×10^−6^, *P*=0.984; lunge-associated: *ŷ*=0.080*x*+0.862, *R*^2^=0.04, *P*=0.091; Fig. 3B).

The mean percentage change in swimming speed (Δ*U*) was found to be lower for routine swimming (11.79±1.314%) than for lunge-associated swimming (24.02±2.162%). Among the six species, the blue whale displayed the highest Δ*U* as a value and as a percentage for both routine (0.15±0.027 m s^−1^; 16.04±0.875%) and lunge-associated swimming (0.80±0.038 m s^−1^; 32.09±1.369%). The other five species did not display a consistent order for Δ*U* as a value or as a percentage or between routine and lunge-associated swimming. For routine swimming, the fin whale had the second highest Δ*U* as a percentage (15.06±1.256%) and the only negative mean value (−0.07±0.030 m s^−1^); the humpback, Antarctic minke and sei whales had similar Δ*U* as a percentage (humpback: 11.60±0.900%; Antarctic minke: 10.89±0.473%; sei: 9.59%), with the humpback and sei whales having slightly higher values (humpback: 0.08±0.012 m s^−1^; sei: 0.09 m s^−1^) than the Antarctic minke whale (0.06±0.009 m s^−1^); and the Bryde's whale had the lowest Δ*U* as a value and as a percentage (0.05±0.028 m s^−1^; 7.62±0.153%). For lunge-associated swimming, the Bryde's and humpback whales had the second and third highest Δ*U* values (Bryde's: 0.53±0.134 m s^−1^; humpback: 0.46±0.055 m s^−1^) but a flipped order for the percentages (Bryde's: 25.79±5.881%; humpback: 26.68±1.899%); the fin whale had the fourth largest Δ*U* as both a value and a percentage (0.40±0.412 m s^−1^; 22.43±0.393%); and the Antarctic minke and sei whales had very similar Δ*U* values (Antarctic minke: 0.36±0.068 m s^−1^; sei: 0.37 m s^−1^), with the Antarctic minke whale having a higher percentage (19.80±1.272%) than the sei whale (17.33%). These Δ*U* values, in turn, yielded values of the unsteady-motion correction to *C*_D,avg_ (i.e. the second term on the right-hand side of Eqn 8a), estimated at 59.10±23.57% for the humpback whale, 28.5±5.48% for the blue whale, 15.14±22.39% for the Antarctic minke whale, 8.98% for the sei whale, 5.16±1.99% for the Bryde's whale and 2.48±1.46% for the fin whale.

All species-level means (±s.e.m.) for each of our measured kinematic and morphometric variables are given in Table 1. The equations and statistics pertaining to our models are given in Table 2.

**Table 2.**
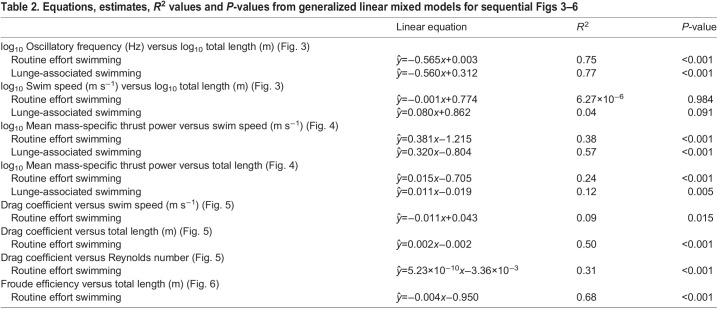
Equations, estimates, *R*^2^ values and *P*-values from generalized linear mixed models for sequential Figs 3–6

### Mass-specific mechanical thrust power output

Among the three species with a large amount of data in our dataset (humpback, blue and Antarctic minke whales) and during routine swimming, the humpback whale had the lowest mean mass-specific thrust power output (0.27±0.023 W kg^−1^), with the Antarctic minke whale having a slightly higher value (0.31±0.023 W kg^−1^) and the blue whale having the highest value (0.42±0.024 W kg^−1^). The Bryde's (0.44±0.167 W kg^−1^), sei (0.48 W kg^−1^) and fin whales (0.64±0.229 W kg^−1^) each had higher values. During lunge-associated swimming, the sei whale had the lowest value (0.87 W kg^−1^), with the Antarctic minke (1.23±0.150 W kg^−1^) and humpback whales (1.30±0.138 W kg^−1^) having similar values and the blue (1.85±0.111 W kg^−1^), fin (2.04±1.293 W kg^−1^) and Bryde's whales (3.03±0.527 W kg^−1^) all having higher values.

**Fig. 4.**
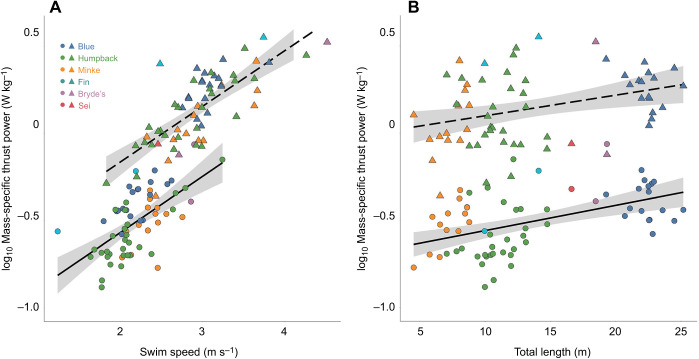
**Comparison of swim speed and total body length against mass-specific thrust power for two swimming modes.** Linear regressions showing (A) swim speed (m s^−1^) and (B) total body length (m) versus the log_10_ of mass-specific thrust power output (W kg^−1^) for both routine swimming (solid line) and lunge-associated swimming (dashed line). Each point corresponds to the mean value for a single individual whale and a single swimming mode (circle: routine; triangle: lunge-associated).

Mean mass-specific thrust power output increased with the transition from routine to lunge-associated swimming modes (Fig. 4), and to values in agreement with an alternative approach based on the work-energy theorem (Potvin et al., 2021). There was a positive effect of swimming speed on mass-specific thrust power output during both routine and lunge-associated swimming (routine: *ŷ*=0.381*x*−1.215, *R*^2^=0.38, *P*<0.001; lunge-associated: *ŷ*=0.320*x*−0.804, *R*^2^=0.57, *P*<0.001; Fig. 4A). We also found that mean mass-specific thrust power output increased with body length for both routine (*ŷ*=0.015*x*−0.705, *R*^2^=0.24, *P*<0.001) and lunge-associated swimming (*ŷ*=0.011*x*−0.019, *R*^2^=0.12, *P*=0.005; Fig. 4B). The species-level means (±s.e.m.) for each of our measured hydrodynamic parameters are given in Table 3.

**Table 3.**
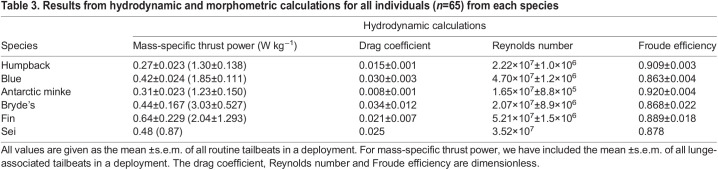
Results from hydrodynamic and morphometric calculations for all individuals (*n*=65) from each species

### Drag coefficient

**Fig. 5.**
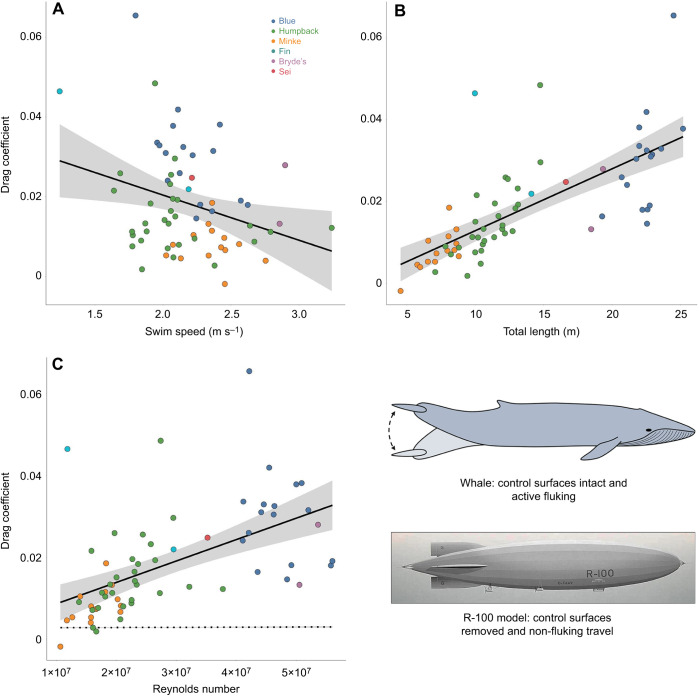
**Comparison of swim speed, total body length and Reynolds number against drag coefficient for routine swimming.** Linear regressions showing (A) swim speed (m s^−1^), (B) total body length (m) and (C) Reynolds number (dimensionless) versus the drag coefficient (dimensionless) for routine swimming (solid line). Each point corresponds to the mean value for a single individual whale and a single swimming mode (circle: routine; triangle: lunge-associated). Dotted line shown in C is a linear regression of Reynolds number versus drag coefficient for a simple rigid-body model comparison using equations derived from Hoerner (1965). Illustration shows a swimming blue whale and image shows an R-100 rigid body as visual representations of the data shown in C.

Among humpback, blue and Antarctic minke whales, the Antarctic minke whale had the lowest mean drag coefficient (0.008±0.001), with the humpback whale slightly higher (0.0015±0.001) and the blue whale having the highest value (0.030±0.003). We found that the drag coefficient for routine swimming decreased with increasing swim speed (routine: *ŷ*=−0.011*x*+0.043, *R*^2^=0.09, *P*=0.015; Fig. 5A). Conversely, the drag coefficient increased for routine swimming with increasing total body length (routine: *ŷ*=0.002*x*−0.002, *R*^2^=0.50, *P*<0.001; Fig. 5B).

We found that the drag coefficient increased significantly with Reynolds number (routine: *ŷ*=5.23×10^−10^*x*−3.36×10^−3^, *R*^2^=0.31, *P*<0.001; Fig. 5C). In comparison with the R-100 rigid-hulled airship model, all species displayed higher drag coefficients by an approximate factor of 3 for the Antarctic minke whale and as high as 14 for the Bryde's whale (Fig. 5C), which are consistent with the discrepancies found among odontocetes (Fish, 1993, 1998; Fish et al., 2014).

### Froude efficiency

Of the three species with a large quantity of data in our dataset (humpback, blue and Antarctic minke whales), the Antarctic minke whale had the highest mean Froude efficiency during routine swimming (0.920±0.004), with the humpback whale having a lower mean value (0.909±0.003) and the blue whale having the lowest mean value (0.863±0.004). The mean values for the Bryde's (0.868±0.022), sei (0.878) and fin whales (0.889±0.018) were all near the low end of the range.

**Fig. 6.**
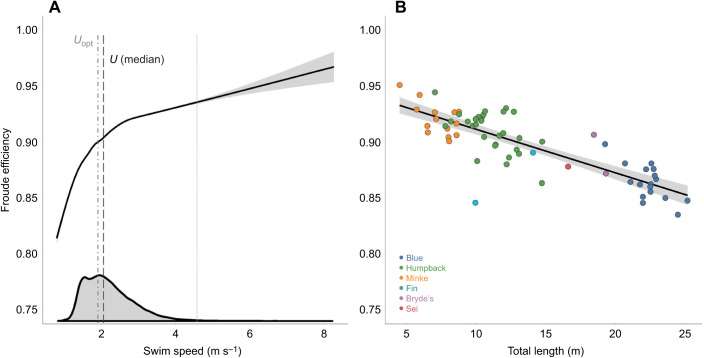
**Comparison of swim speed and total body length against Froude efficiency.** Curved fit lines showing (A) swim speed (m s^−1^) and linear regression showing (B) total body length (m) versus Froude efficiency (dimensionless) for routine swimming (solid line). Curved fit line shown in A is based upon each individual tailbeat measurement for all species combined and shows the plateau in Froude efficiency that occurs at 2–2.5 m s^−1^. Vertical black dashed line in A denotes the median routine swimming speed across all species (2.06 m s^−1^). Vertical gray dot-dashed line in A denotes the optimal swimming speed (*U*_opt_; 1.97 m s^−1^) calculated by Gough et al. (2019). Vertical gray dotted line at 4.5 m s^−1^ in A denotes the 99th percentile, with only 1% of the data falling to the right of the line. Each point in B corresponds to the mean value for a single individual whale. Gray density plot along *x*-axis of A shows the density of swim speeds for all species combined.

**Fig. 7.**
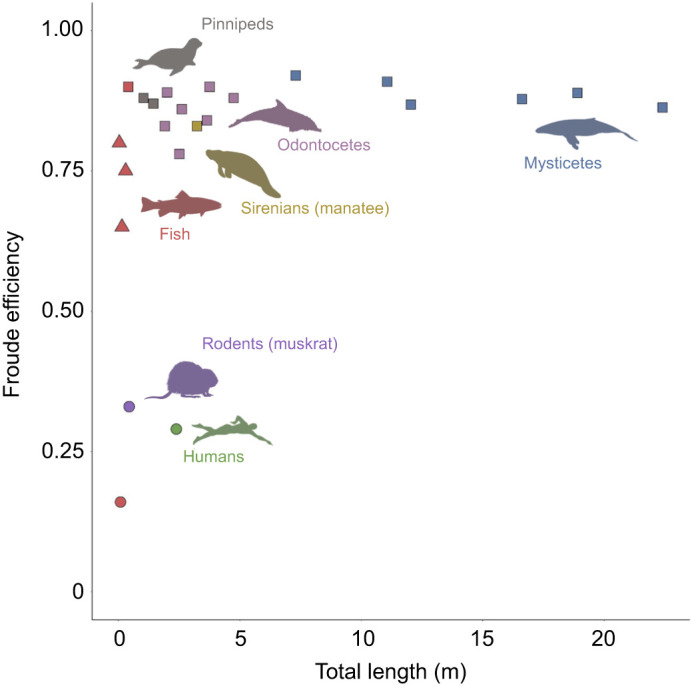
**Froude efficiency versus total body length (m) for species from different morphological and taxonomic groups that use different swimming modes.** The values for mysticete cetaceans are the mean species-level data from our present study. Silhouettes correspond to each group by rough position and color. Circle: drag-based paddling; triangle: undulatory swimming; square: oscillatory swimming.

Mean Froude efficiency increased with increasing swimming speed up to an approximate plateau at ∼3 m s^−1^ (Fig. 6A). In contrast, mean Froude efficiency decreased with increasing body length (routine: *ŷ*=−0.004*x*−0.950, *R*^2^=0.68, *P*<0.001; Fig. 6B). As compared with prior studies, our results demonstrate that, regardless of body size, rorqual whales demonstrate high efficiency (>75%) comparable to other oscillatory swimmers (Fig. 7). Sub-carangiform, undulatory swimmers such as the rainbow trout (*Oncorhynchus mykiss*) are slightly lower (∼60–80%) and drag-based swimmers, such as the muskrat and human, have much lower Froude efficiencies (∼20–35%) (Fig. 7). Table S3 gives additional information about each literature-based mean Froude efficiency value.

## DISCUSSION

Many previous studies that have quantified the kinematics and hydrodynamics of cetacean swimming have used captive animals that can be measured reliably from a stable reference position (Fish, 1993, 1998; Rohr and Fish, 2004). By comparison, the present study is a first approximation for many of the same kinematic variables of much larger species in their natural environment. Several parameters, such as the angle of attack of the flukes relative to the body or the amplitude of heave, are still generally unknown (except in rare circumstances, see Gough et al., 2019), so we supplemented our empirical data with validated estimates for these unknown variables (Bainbridge, 1958; Fish, 1998). The angle of attack of the fluke has been found to change with speed over a range from 20 to 40 deg, so we used 30 deg as an average value (Fish, 1998). Amplitude of heave has been reliably measured as one-fifth of body length and remains constant across swimming speeds and body size (Bainbridge, 1958; Fish, 1998). Our combination of empirical measurements and reliable estimates allowed us to quantify hydrodynamic and kinematic aspects of mysticete swimming using a numerical computation based on unsteady lifting-surface theory and derived by Chopra and Kambe (1977), which has also been validated for odontocetes by Fish (1998). The similarity between our methods and those of previous studies extends our ability to compare swimming performance across vast body size ranges.

### Oscillatory frequency and swimming speed

Our results illustrate that the transition from routine to lunge-associated swimming predictably results in increased oscillatory frequencies and swimming speeds as the animal prepares for a lunge (Fig. 3) (Goldbogen et al., 2011; Cade et al., 2016). Gough et al. (2019) found that the oscillatory frequency decreases with increasing body size to the power of −0.53, and with a more robust data set we have found similar scaling exponents of −0.565 and −0.560 for routine and lunge-associated swimming, respectively. For swimming speed, we again found similar results to Gough et al. (2019) with swimming speed remaining consistent at ∼2 m s^−1^. For both oscillatory frequency and swimming speed, the scaling exponents for routine and lunge-associated swim efforts were nearly identical, with a difference of 0.005 for oscillatory frequency and a difference of 0.081 for swimming speed. This suggests that, regardless of body size, mysticetes prepare for a feeding lunge through similar kinematic pathways that include a consistent increase in both oscillatory frequency and swimming speed. These results for oscillatory frequency and swimming speed align with previous results for fish and odontocetes that have shown that swimming speed is heavily modulated by oscillatory frequency (Bainbridge, 1958; Fish, 1998; Gough et al., 2019).

### Mean mass-specific thrust

Thrust generation is a fundamental aspect of any swimming mode and the achievable thrust for a swimming animal has a direct impact on its maximum swimming speed and, subsequently, the types and quantities of prey that it can capture (Fish, 1998; Potvin et al., 2009; Cade et al., 2020). Hydrodynamic theory states that thrust should increase with the square of velocity (Webb, 1975; Vogel, 1994). Thrust from an oscillating hydrofoil will further increase the thrust of a system by three to five times (Lighthill, 1971; Liu et al., 1997; Anderson et al., 2001; Fish et al., 2014). Although this theory holds for animals of similar sizes, we found it advantageous to measure the mass-specific thrust to make comparisons between mysticetes and other cetaceans that vary across a wide range of body sizes.

For cetaceans, high mass-specific thrust allows odontocetes to capture fast-moving, individual fish (Maresh et al., 2004), and allows mysticetes to achieve high speeds during feeding lunges to offset the deceleration during prey engulfment as well as the potential escape response of different prey types (Cade et al., 2016, 2020). Fish (1998) measured the mass-specific thrust of odontocete species and found maximum mass-specific thrust values of 22.5 and 23.7 W kg^−1^ for *Pseudorca crassidens* and *Tursiops truncatus*, respectively. The maximum mass-specific thrust value for a mysticete (Bryde's) swimming at 6.3 m s^−1^ (lunge-associated) in our study was 16 W kg^−1^, but mass-specific thrust values at the species-level averaged between 0.87 and 3.03 W kg^−1^ for lunge-associated swimming and between 0.27 and 0.64 W kg^−1^ for routine swimming, which were one to two orders of magnitude lower (Fig. 4A, Table 3). These results suggest that mysticetes typically maintain low average mass-specific thrust values in accordance with their relatively steady swimming speeds (∼1.5–2.5 m s^−1^), but that they can attain extremely high mass-specific thrust power output when properly motivated. Swimming speeds higher than those found in our dataset have also been found for humpback whales (up to ∼9 m s^−1^; Tomilin, 1957; Segre et al., 2020), indicating that they could be producing mass-specific thrust values on par with odontocetes during fast maneuvers such as surface breaches.

Our comparisons of speed-matched mass-specific thrust output between routine swimming and lunges suggest that whales likely alter oscillatory frequency in order to generate greater thrust during feeding (Gough et al., 2019). Mass-specific thrust power at a routine swimming speed (∼1.5–2.5 m s^−1^) results in a low propulsive energy cost (Gough et al., 2019). The relative similarity of the mass-specific thrust increase (∼two-fold) from routine to a lunge-feeding effort across our range in body size suggests that all of the large whales studied are preparing for a lunge in similar ways. Field data (Cade et al., 2020) and hydrodynamic models (Potvin et al., 2009, 2020) suggest that the whales begin lunges at high speeds (3.5–5 m s^−1^) in order to overcome heightened drag during engulfment, and krill feeders usually move through the prey patch on momentum (Potvin et al., 2009).

Focusing more heavily on the relationship between mass-specific thrust generation and body size, our results diverge slightly from previous estimates. Fish (1998) determined that mass-specific thrust and body size have no relationship. Hill (1950) considered that for similar animals, the maximum power generated during a steady effort would increase not directly with the weight (*W*), but rather with *W*^0.73^. As a result, we expected that mass-specific thrust would decrease proportionately with increasing body size. Instead, we found that mass-specific thrust increases as body length increases (Fig. 4). This relationship could result from the higher oscillatory frequencies with larger body sizes that Gough et al. (2019) and our present study found in contrast to previous expectations of oscillatory frequency (Hill, 1950; Sato et al., 2007). For the relationship between oscillatory frequency and body length, Sato et al. (2007) found a more extreme allometric scaling exponent (approximately −1.0), whereas Gough et al. (2019) and the present study found an exponent of approximately −0.5, suggesting a less extreme decrease in oscillatory frequency with increasing body length.

### Drag coefficient versus Reynolds number

In comparison to our tagged animals, Hoerner's R-100 airship model used for computational analysis did not include control surfaces (flippers or flukes). Instead, the approximated environment around the airship was determined using wind tunnel test data (Hoerner, 1965; Blevins, 1984). These modeled values suggest that for an Antarctic minke whale (∼5 m), the drag coefficients for fluking should be roughly three times higher than for non-fluking and gliding. But the difference between these coefficients should increase for larger animals, culminating in a 10-fold difference for a blue whale (∼22 m) (Fig. 5B). Other studies predicted similar increases in the drag coefficient, with Lighthill (1971) first noticing a discrepancy between the expected drag coefficient based on Hoerner's model and the observed values for swimming fish, but his conclusions did not account for changing Reynolds numbers and were based upon animals swimming at Reynolds numbers of ∼10^5^, whereas large cetaceans are routinely swimming at values of ∼10^7^. Fish (1993) included a variety of species and groups and found higher drag coefficient values for swimming animals as compared with model estimates, but they did not find an increase with increasing Reynolds number as we have for larger cetaceans (Fig. 5C). Fish (1998) analyzed how the drag coefficient might vary with Reynolds number among four species of odontocetes and found that the drag coefficient should decrease with increasing Reynolds number.

For mysticetes, we found a negative relationship between the drag coefficient and the swimming speed as well as a positive relationship between the drag coefficient and body length (Fig. 5A,B). Reynolds number is affected by both the swimming speed and the body length of an animal, so we believe that the impact of body size between individuals is more extreme than the impact of swimming speed within individuals, resulting in a net positive impact of Reynolds number on drag coefficient (Fig. 5C). The effects of swimming speed on drag coefficient have been determined previously by Fish (1998) for a group of odontocetes, but ours is the first study that includes a large enough body size range to be able to parse out the effect of body size on both Reynolds number and drag coefficient.

### Froude efficiency versus swimming velocity

Optimal locomotor speeds have been demonstrated for runners, flyers and swimmers (e.g. Tucker, 1968; Webb, 1975; Hoyt and Taylor, 1981; Watanabe et al., 2011). The cost of transport (COT) has been used as the metabolic proxy that is inversely related to the Froude efficiency (Williams et al., 1993; Fish, 2000), and Yazdi et al. (1999) found that the minimum COT for the bottlenose dolphin (*Tursiops truncatus*) occurred at a swimming speed of 2.5 m s^−1^, which coincided with the routine swimming speeds in wild populations. Similarly, gray whales (*Eschrichtius robustus*) and Antarctic minke whales cruise at the speed of the lowest COT (Sumich, 1983; Blix and Folkow, 1995). The minimum COT for the gray whale corresponded to the swimming velocity (2.0–2.5 m s^−1^) of migrations (Wyrick, 1954; Williamson, 1972; Sumich, 1983). Antarctic minke whales, however, were determined to have a minimum COT at the maximum cruising velocity of 3.25 m s^−1^ (Blix and Folkow, 1995), which was 37% higher than the average routine swimming speed (2.35 m s^−1^) in the present study. This average velocity was within the range of swimming velocities (1.5–2.6 m s^−1^) for migrating Antarctic minke whales (Williamson, 1972), a range that accounted for 56.5% of the routine swimming speed measurements for Antarctic minke whales in our dataset. The average routine swimming velocities for blue (2.20 m s^−1^) and humpback whales (2.09 m s^−1^) also fell within ranges of migratory velocities of 1.5–3.1 m s^−1^ (Williamson, 1972) and 1.1–4.0 m s^−1^ (Chittleborough, 1953; Williamson, 1972), respectively. These ranges accounted for 67.1% of the routine swimming speed measurements for the blue whales and 99.0% of the same measurements for the humpback whales in our dataset. The average (2.18 m s^−1^) and median (2.06 m s^−1^) routine swimming speed that we found among all species fell near the center of these migratory speed ranges and aligned closely with the optimal swimming speed (*U*_opt_; 1.97 m s^−1^) predicted by Gough et al. (2019) (Fig. 6A).

Only 1% of our speed measures fell above 4.5 m s^−1^, meaning our ability to predict Froude efficiency at these high speeds is limited. The significantly unsteady nature of lunge-associated swimming also meant that we could not include that swimming style in our analysis of Froude efficiency. Our results for routine swimming below 4.5 m s^−1^ show that Froude efficiency increases rapidly below ∼2 m s^−1^ and plateaus, which broadly agrees with the results from Fish (1998) for odontocetes. The position of the plateau relative to the average routine swimming speed and the optimal swimming speed from Gough et al. (2019) suggest that these species are simultaneously minimizing their swimming speed and maintaining high Froude efficiency along the plateau (Fig. 6A).

### Froude efficiency versus body size

In the present study, Froude efficiency relates to the amount of mechanical work the animal does to propel itself forward. Previous research has shown that Froude efficiency would remain constant or slightly increase with increasing body size (Fish, 1998). However, we found that Froude efficiency decreases with increasing body size among rorquals (see Fig. 7B). The mechanistic explanation of this finding is that larger individuals have a slightly increased thrust generation but a greatly increased drag coefficient (Figs 4 and 5), thus resulting in a lower Froude efficiency, because more energy may be required to overcome drag and achieve equivalent locomotor performance.

Our analyses suggest that size is an important determinant of swimming efficiency in rorquals. Balaenopteridae exhibit a size range than spans an order of magnitude in body mass, from Antarctic minke whales to blue whales (Lockyer, 1976). The scale of these ocean giants necessitates the use of oscillatory lift-based swimming as an effective propulsive mechanism for high-speed swimming at high Reynolds numbers (Webb and De Buffrénil, 1990; Fish, 2020). Interestingly, in parallel with the trend of maximum speed in which intermediately sized animals (∼250 kg; the approximate size of a common bottlenose dolphin) exhibited the highest performance with lower maximum speeds for small and large animals, it was found for whales that Froude efficiency, another locomotor performance variable, decreased above and below a different and larger optimal size, roughly between a killer whale and a minke whale (Hirt et al., 2017) (Fig. 7, Table S3).

### Conclusions

The thrust power and drag coefficient produced by rorquals during routine swimming increased with body size. However, the Froude efficiency was found to decrease with increasing body size. These conclusions ran counter to our expectations of the swimming performance of cruising rorquals. During foraging, these animals swim over a wider speed range and produce greater maximum thrust than exhibited at routine speeds. This difference is predictable owing to a higher oscillatory frequency during foraging bouts in which the whale beats its tail faster to accelerate to the high speeds necessary to overcome the increased drag as the mouth opens during engulfment and prey capture. Our results quantify the fine-scale hydrodynamics that underlie these energetic differences between routine swimming and energetically expensive foraging. In addition, we show that large whales – across a range of body sizes – can modulate their swimming kinematics to optimize energy use, but might experience a reduced energy economy as Froude efficiency decreases with increasing body size.

## Supplementary Material

Supplementary information
