# Supplementary material for: Scaling of oscillatory kinematics and Froude efficiency in baleen whales
Source: J Exp Biol. 2021 Jul 9;224(13):jeb237586. doi: 10.1242/jeb.237586 (PMC8317509; doi:10.1242/jeb.237586)
Supplement: Supplementary information [file jexbio-224-237586-s1.pdf]

Table S1. All symbols and corresponding definitions (with units) used throughout the manuscript. Symbols are presented in the order in which they appear in the text.

Symbol	***Definition***
GSD	Ground sampling distance (m)
L_{body}	Body length (m)
n_{pix}	Number of pixels (count)
a	Altitude (m)
l_{foc}	Focal length (mm)
S_w	Sensor width (mm)
P_w	Image resolution width (px)
F_a	Planar fluke area (m^2)
C	Chord length of tail (m)
M_{body}	Body mass (kg)
S_a	Wetted surface area of body (m^2)
U_{avg}	Mean swimming velocity (m s^{-1})
f η	Oscillatory frequency (Hz)
T_{beat}	Duration of a tailbeat (s)
$\bar{P}_T$	Mechanical thrust power (W)
C_D	Coefficient of drag (dimensionless)
	Froude efficiency (dimensionless)
σ	Reduced frequency (dimensionless)
ω	Angular frequency of fluking (Hz)
θ	Feathering parameter (dimensionless)
α	Angle of attack of flukes (degrees)
h	Heaving amplitude (m)
C_T	Coefficient of thrust (dimensionless)
$\bar{T}$	Mean thrust force (N)
ρ	Density of seawater (Kg m^{-3})
$\bar{D}$	Mean drag force (N)
$\bar{a}$	Mean acceleration (m s^{-2})
U_f	Final tailbeat swimming speed (m s^{-1})
U_i	Initial tailbeat swimming speed (m s^{-1})
ΔU	Change in tailbeat swimming speed (m s^{-1})
k_{added}	Shape drag correction factor (dimensionless)
$C_D^{routine}$	Mean drag coefficient for all routine tailbeats from a single whale (dimensionless)
$\bar{P}_T^{lunge}$	Thrust power for a lunge-associated tailbeat (W)
C_D^{mod}	Drag coefficient from rigid airship model
W_{max}	Maximum body diameter (m)
Re	Reynold's number (dimensionless)
	Kinematic viscosity (m^2 s^{-1})

Table S2. Equations used to calculate the wetted surface area of each species as well as literature sources.

Species	Source	Body Length (m)	Surface Area (m^2)	Surface Area Equation
Humpback	CFD model – Kennedy (2021)	14.78	82	$S_a = 5.55 \times L_{body}$
Blue	Kermack, 1948	25.91	175.59	$S_a = 6.78 \times L_{body}$
Antarctic Minke	CFD model – Kennedy (2021)	8	28	$S_a = 3.50 \times L_{body}$
Bryde's	Fish (pers comm.)	-	-	$S_a = 0.43185 \times L_{body}^{1.9103}$
Fin	Parry, 1949	19.8	137	$S_a = 5.81 \times L_{body}$
	Kermack, 1948	20.12	115.11	
	Kermack, 1948	21.1	126.07	
	Bose and Lien, 1989	14.5	67.35	
Sei	Fish (pers comm.)	-	-	$S_a = 0.43185 \times L_{body}^{1.9103}$

Journal of Experimental Biology • Supplementary information

Table S3. Froude efficiency and metadata collected from various sources for the creation of figure 7.

Species	Swim Speed (m s^{-1}) or (bl s^{-1})*	Total Length (m)	Froude Efficiency	Source(s)
Homo sapien *Human (Female)*	0.95	2.38	0.29	von Loebbecke et al., 2009
Ondatra zibethicus *Muskrat*	0.75	0.44	0.33	Fish, 1984
Pterophyllum eimekei *Freshwater Angelfish*	0.04	0.08	0.16	Blake, 1979; Blake, 1980
Danio rerio *Zebra Danio*	Multiple	0.0315	0.80	McCutchen, 1975
Cymatogaster aggregata *Shiner Perch*	0.57	0.143	0.65	Webb, 1975
Oncorhynchus mykiss *Rainbow Trout*	U_{crit}	0.293	0.75	Webb, 1975
Euthynnus affinis *Mackerel Tuna (Kawakawa)*	1.52	0.40	0.90	Magnuson, 1978
Pusa hispida *Ringed Seal*	0.75	1.03	0.88	Fish et al., 1988
Pagophilus groenlandicus *Harp Seal*	1.04	1.43	0.87	Fish et al., 1988
Trichechus manatus *American Manatee*	0.30*	3.23	0.83	Kojeszewski and Fish, 2007
Delphinapterus leucas *Beluga Whale*	3.00	3.64	0.84	Fish 1998
Lagenorhynchus obliquidens *Pacific White-Sided Dolphin*	5.30	2.00	0.89	Webb, 1975; Yates, 1983; Blickhan and Cheng, 1994

Journal of Experimental Biology • Supplementary information

Orcinus orca *Killer Whale*	6.50	4.74	0.88	Fish, 1998
Pseudorca crassidens *False Killer Whale*	3.80	3.75	0.90	Fish, 1998
Sotalia guianensis *Guiana Dolphin*	2.40	1.90	0.83	Blickhan and Cheng, 1994
Tursiops truncatus *Common Bottlenose Dolphin*	2.40[1], 3.80[2]	2.50[1], 2.61[2]	0.78[1], 0.86[2]	Blickhan and Cheng, 1994[1]; Fish, 1998[2]
Megaptera Novaeangliae *Humpback Whale*	2.09 ± 0.066 (Routine Effort Swimming)	11.06 ± 0.35	0.909 ± 0.003	Current Study
Balaenoptera musculus *Blue Whale*	2.20 ± 0.054 (Routine Effort Swimming)	22.41 ± 0.33	0.863 ± 0.004	Current Study
Balaenoptera bonaerensis *Antarctic Minke Whale*	2.35 ± 0.052 (Routine Effort Swimming)	7.30 ± 0.34	0.920 ± 0.004	Current Study
Balaenoptera brydei *Bryde's Whale*	1.71 ± 0.47 (Routine Effort Swimming)	12.04 ± 2.07	0.868 ± 0.022	Current Study
Balaenoptera physalus *Fin Whale*	2.88 ± 0.020 (Routine Effort Swimming)	18.90 ± 0.43	0.889 ± 0.018	Current Study
Balaenoptera borealis *Sei Whale*	2.21 (Routine Effort Swimming)	16.62	0.878	Current Study

Journal of Experimental Biology • Supplementary information
